# Coexpression network analysis of the adult brain sheds light on the pathogenic mechanism of *DDR1* in schizophrenia and bipolar disorder

**DOI:** 10.1038/s41398-024-02823-0

**Published:** 2024-02-23

**Authors:** Selena Aranda, Gerard Muntané, Elisabet Vilella

**Affiliations:** 1 Institut d’Investigació Sanitària Pere Virgili-CERCA, Reus, Spain; 2 Hospital Universitari Institut Pere Mata, Reus, Spain; 3 Universitat Rovira i Virgili, Reus, Spain; 4 Centro de Investigación Biomédica en Red en Salud Mental (CIBERSAM)-Instituto de Salud Carlos III, Madrid, Spain; 5 Institut de Biologia Evolutiva (UPF-CSIC), Departament de Medicina i Ciències de la Vida (MELIS), Universitat Pompeu Fabra, Barcelona, Spain

**Keywords:** Biomarkers, Bipolar disorder, Schizophrenia

## Abstract

*DDR1* has been linked to schizophrenia (SCZ) and bipolar disorder (BD) in association studies. *DDR1* encodes 58 distinct transcripts, which can be translated into five isoforms (*DDR1*a-e) and are expressed in the brain. However, the transcripts expressed in each brain cell type, their functions and their involvement in SCZ and BD remain unknown. Here, to infer the processes in which *DDR1* transcripts are involved, we used transcriptomic data from the human brain dorsolateral prefrontal cortex of healthy controls (*N* = 936) and performed weighted gene coexpression network analysis followed by enrichment analyses. Then, to explore the involvement of *DDR1* transcripts in SCZ (*N* = 563) and BD (*N* = 222), we studied the association of coexpression modules with disease and performed differential expression and transcript significance analyses. Some *DDR1* transcripts were distributed across five coexpression modules identified in healthy controls (M_HC_). M_HC_1 and M_HC_2 were enriched in the cell cycle and proliferation of astrocytes and OPCs; M_HC_3 and M_HC_4 were enriched in oligodendrocyte differentiation and myelination; and M_HC_5 was enriched in neurons and synaptic transmission. Most of the *DDR1* transcripts associated with SCZ and BD pertained to M_HC_1 and M_HC_2. Altogether, our results suggest that *DDR1* expression might be altered in SCZ and BD via the proliferation of astrocytes and OPCs, suggesting that these processes are relevant in psychiatric disorders.

## Introduction

*DDR1* is a pleiotropic membrane-anchored tyrosine kinase receptor that is expressed in multiple tissues, including the brain [1, 2]. *DDR1* gene variants were found to be associated with schizophrenia (SCZ) in candidate gene studies [3–5]. In addition, genome-wide association studies found associations between *DDR1* SNPs and SCZ [6, 7] and bipolar disorder (BD) [8]. However, they did not include the *DDR1* locus in the final analyses because it falls inside of a linkage disequilibrium region with the highest SCZ-associated locus; therefore, these results can be found only in the supplementary materials.

In situ hybridization studies in mice demonstrated that *DDR1* is expressed in areas of neurogenesis prenatally, while it overlaps with the dynamics of the myelination process during the postnatal period [9]. In the adult mouse brain, *DDR1* mRNA expression was first detected in glia [10], and later, single-cell transcriptome profile analysis showed that *DDR1* is mainly expressed in cells of the oligodendrocyte lineage and to a lesser extent in neurons and astrocytes, while its expression in microglia and endothelial cells was almost undetectable [11]. In the adult human brain, the presence of *DDR1* is also detected in myelin and in the soma of oligodendrocytes, astrocytes and endothelial cells [12].

Alternative splicing of *DDR1* produces 58 transcripts, some of which encode one of the five isoforms known to date, named *DDR1*a-e [13–15]. Each isoform may have different functions. For instance, the upregulation of two different transcripts (encoding *DDR1*a and *DDR1*b) after irradiation of in vitro cultured astrocytes suggests their involvement in DNA repair, checkpoint signaling pathways and apoptosis [16, 17]. Additionally, *DDR1*a was shown to promote the migration of leukocytes [18] and cell invasion and adhesion in gliomas [19]. *DDR1*c mRNA expression in the human brain correlates with the expression of *OLIG2* and *MAG*, two oligodendrocyte protein markers [20], and is upregulated in brain dorsolateral prefrontal cortex (DLPFC) tissue from patients with SCZ compared to healthy controls (HCs) [21].

Studies of *DDR1* expression in the human brain are scarce [22], but data from public repositories show that *DDR1* expression decreases with age [23]. Studying coexpression networks during human brain development, we previously described that *DDR1* was coexpressed mainly with oligodendrocyte-related genes in the postnatal and early adulthood periods (between 0 and 40 years old) and with astrocyte- and type 2 microglia-related genes in the prenatal period and late adulthood ( > 40 years old) [23]. However, a comprehensive description of each *DDR1* transcript coexpression network in human brain tissue and, especially, in psychiatric disorders has not yet been performed.

Here, we used bulk expression data from the human DLPFC to (1) build gene coexpression modules and assess the cell type and biological process enrichments for modules containing *DDR1* transcripts, (2) evaluate the association of these coexpression modules with SCZ and BD and (3) test the differential expression of *DDR1* transcripts in patients with SCZ or BD with respect to HCs.

## Materials and methods

### Sample data

Data for this publication were obtained from the NIMH Repository and Genomics Resource, a centralized national biorepository for genetic studies of psychiatric disorders. We retrieved the gene and transcript-level counts from DLPFC samples obtained by RNA-Seq by Expectation-Maximization (RSEM) and corresponding clinical information from HCs, patients with SCZ and patients with BD from 6 different collections of the PsychENCODE project: BrainGVEX, BrainSpan, CMC, BipSeq, LIBD and CMC-HBCC. We removed samples with a diagnosis other than SCZ or BD (*N* = 9) or without age data (*N* = 23) to ultimately include 936 HCs, 563 patients with SCZ and 222 patients with BD in the analyses. We tested for differences in sex, age of death and ethnicity among diagnostic groups in each collection and in the whole sample. The chi-squared test was used to compare categorical variables, the Mann‒Whitney test was used to compare continuous variables between two different diagnostic groups, and the Kruskal‒Wallis test was used to compare continuous variables among three different diagnostic groups (Table 1). Lowly expressed genes and transcripts were filtered out using the filterByExpr() function of the edgeR package v3.40.1 [24] to retain genes and transcripts with at least 10 counts per million in 70% of the samples of the smallest group, as previously described [25], and counts from 22548 genes and 81950 transcripts per individual remained. Of note, 43 *DDR1* transcripts were excluded from the analysis, and 15 were included: ENST00000376567, ENST00000376570, ENST00000376569 and ENST00000418800 encode *DDR1*a; ENST00000324771, ENST00000376568 and ENST00000452441 encode *DDR1*b; ENST00000513240 encodes *DDR1*c; ENST00000376567 encodes *DDR1*d; ENST00000508312 encodes *DDR1*e; ENST00000428153, ENST00000460944, ENST00000484556 and ENST00000446312 encode other *DDR1* protein isoforms (not *DDR1*a-e); and ENST00000508472 is a noncoding transcript. We downloaded genotype data (7.5 million variants) for 68 HC samples from the LIBD collection (Table 1). More detailed information about each collection is reported in the Expression data section of the Supplementary Material.

**Table 1:** Sample description.

Study	Diagnostic	*N*	Sex (%male/%female)		Age of death		Ethnicity (%)	
			Proportion	P*	Mean	P*	Proportion	P*
Brain GVEX	HC	259	0.65/0.35	0.034	72.61 ± 18.41		0.98 CAU, 0.008 AS, 0.008 HISP	0.331
	SCZ	94	0.71/0.29		42.55 ± 10.36	2.2E-16	0.98 CAU, 0.01AA, 0.01 Nonwhite	
	BD	73	0.52/0.48		43.99 ± 11.53		0.97 CAU, 0.015 AS, 0.015 Nonwhite	
BrainSpan	HC	22	0.60/0.40	-	13.18 ± 12.22	-	0.50 AA, 0.45 CAU, 0.45 HISP	-
CMC	HC	284	0.57/0.43	0.198	65.08 ± 19.03	1.0E-08	0.76 CAU, 0.16 AA, 0.07 HISP, 0.01 AS	0.019
	SCZ	264	0.64/0.36		68.46 ± 16.49		0.83 CAU, 0.15 AA, 0.019 HISP, 0.004 AS	
	BD	47	0.55/0.45		50.72 ± 15.12		0.96 CAU, 0.02 AA, 0.02 HISP	
BipSeq	BD	32	0.44/0.56	-	47.56 ± 13.85	-	0.91 CAU, 0.03 AA, 0.03AS, 0.03 HISP	-
LIBD	HC	151	0.63/0.37	0.869	27.44 ± 24.79	2.8E-10	0.52 AA, 0.48 CAU	0.00134
	SCZ	108	0.61/0.39		48.93 ± 15.05		0.69 CAU, 0.31 AA	
	HC**	68	0.34/0.66	-	31.91 ± 24.05	-	0.53 AA, 0.47 CAU	-
CMC_HBCC	HC	220	0.72/0.28	0.691	35.29 ± 20.38	3.7E-09	0.55 AA, 0.41 CAU, 0.022 HISP, 0.018 AS	1.7E-08
	SCZ	97	0.68/0.32		49.90 ± 13.71		0.58 AA, 0.36 CAU, 0.03 AS, 0.03 HISP	
	BD	70	0.69/0.31		42.57 ± 14.53		0.83 CAU, 0.11 AA, 0.03 AS, 0.03 HISP	
Whole sample	HC	936	0.64/0.36	0.074	52.87 ± 27.76	3.8E-11	0.69 CAU, 0.27 AA, 0.03 HISP, 0.01 AS	8.5E-10
	SCZ	563	0.65/0.35		57.19 ± 18.39		0.75 CAU, 0.23 AA, 0.014 HISP, 0.007 AS, 0.002 Nonwhite	
	BD	222	0.57/0.43		45.48 ± 13.91		0.91 CAU, 0.045 AA, 0.02 AS, 0.02 HISP, 0.005 Nonwhite	

*p: *p*-value of the Chi-square, Kruskal‒Wallis or Wilcoxon tests.

**68 out of 151 healthy controls of the LIBD collection used in the eQTL analysis.

*AA* African-American, *AS* Asian, *CAU* Caucasian, *HISP* Hispanic.

### Expression matrix building

**Fig. 1:**
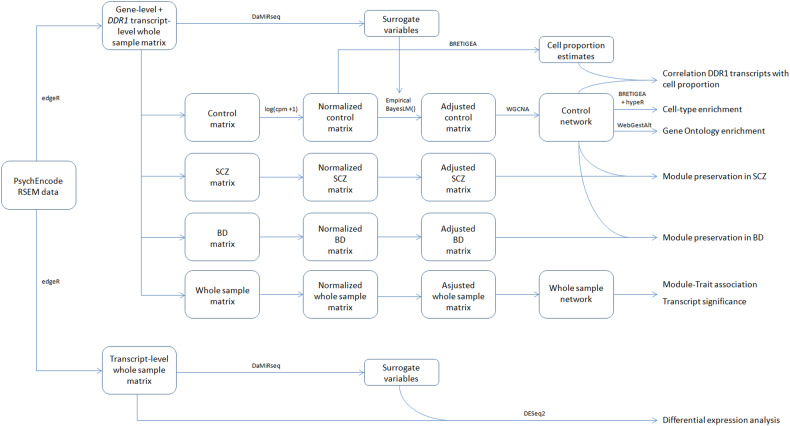
Flow diagram for the data preparation. The PsychEncode RSEM data (leftmost box) correspond to the row data from which gene and transcript level counts were retrieved. Arrows indicate sequencial preparatory steps, boxes display the outputs obtained after each of them and the right column indicates the final analyses performed. The R packages and functions used in each step are also indicated.

Using the readDGE() function of the edgeR package v3.40.1 [24], we built expression matrices from the RSEM files for HCs, patients with SCZ, patients with BD and the whole sample using the gene-level counts of all genes except for *DDR1*, for which we used the transcript-level counts. Counts were normalized to counts per million using the cpm() function of the edgeR package v3.40.1 [24] and log_2_ transformed (Fig. 1). We extracted the principal components (PCs) of the whole sample, plotted PC1 versus PC2 and observed a structure driven mainly by collection (Supplementary Fig. 1A). Furthermore, to remove any variance due to unwanted sources of variation (i.e batches and confounding factors), we used the DaMiRseq R package v2.2.0 [26]. Briefly, the whole-sample expression matrix and clinical information were used to create a summarized object, and the object served as input for DaMiR.SV() function to identify the surrogate variables (SVs) needed to explain 95% of the variance. The number of SVs identified was 4, and all of them correlated with known variables (batch, sex, age of death and ethnicity) but not with diagnostic group (Supplementary Fig. 2), which is a requirement for the use of SVs as covariates [26]. We used the four SVs to adjust the expression matrices using the empiricalBayesLM() function of the WGCNA R package v1.72 [27], which uses Empirical bayes-moderated linear regression to remove unwanted variables (Fig. 1). We plotted PC1 versus PC2 for the new adjusted matrices and observed no sign of structure (Supplementary Fig. 1B).

### Quantification of *DDR1* transcripts

We retrieved the log_2_ transformed counts per million (log_2_CPM) values for each *DDR1* transcript from the adjusted matrices and calculated the mean expression of each *DDR1* transcript independently for HCs, patients with SCZ and patients with BD.

### Expression quantitative trait loci analysis

To study whether SNPs previously reported to explain part of the variation in *DDR1* mRNA expression levels (expression quantitative trait loci (eQTLs) for *DDR1*) in the GTEx portal (https://gtexportal.org/) were eQTLs for individual *DDR1* transcripts, we gathered the whole list of SNPs, performed quality control and filtered out variants that were in high linkage disequilibrium (R^2^ > 0.5) using Plink software v1.9. Ultimately, 146 SNP variants remained. We then selected the corresponding normalized expression data from samples with genotype data available (68 HC samples of the LIBD collection) and performed an eQTL analysis using the GenomicTools R package v0.2.9.7 [28].

### Gene network construction and module detection

Adjusted matrices for HCs and the whole sample were used to generate corresponding unsigned coexpression networks using the WGCNA R package v1.72 (Fig. 1). We chose a soft-threshold power (β) of 9 for both networks to ensure that they were comparable, which allowed us to achieve a scale-free topology fitting index ≥ 0.80 (Supplementary Fig. 3). We set the minimum module size at 30, selected a deep split of 4 and merged highly correlated modules (r^2^ > 0.8). The resulting modules, defined as branches of the network dendrogram, were assigned number labels. We labeled those modules containing *DDR1* transcripts first, and genes or transcripts that did not correspond to any coexpression module were assigned the label M0. To evaluate the importance of each *DDR1* transcript in a module, we calculated their module membership (MM). Briefly, we extracted the principal component of each module, named the module eigengene (ME), which serves as a summary expression measure of the expression profile within a module, and calculated the correlation of the expression of each *DDR1* transcript with the ME of the module in which it was located. *DDR1* transcripts with MM > 0.4 were considered hub transcripts. To enable comparisons between coexpression networks, labels of the whole-sample network were reassigned to match those of the modules with significant overlap in the HC network using the matchLabels() function of the WGCNA R package v1.72, which calculates the overlap of the labels in the whole-sample and HC networks using Fisher’s exact test and relabels whole-sample modules so that each one receives the same label as the control module that it overlaps most with. The HC network (whose modules are named M_HC_) was used to infer the biological importance of each *DDR1* transcript and to test whether the connectivity of the HC network is preserved in SCZ and BD (Fig. 1). The whole sample network (whose modules are named M_WS_) was used to study the correlation of the principal component of each module and the expression of each *DDR1* transcript with the diagnosis of SCZ and BD (Fig. 1). Additionally, the whole sample network was used to study whether the variance explained by the cell type composition of the samples was completely removed by the SVs when building the network. With this aim, we calculated the cell type proportion estimates of the normalized whole sample matrix by using the brainCells() function of the BRETIGEA R package [29] and studied the correlation between the resulting cell type proportion estimates and the MEs of whole sample network.

### Cell-type enrichment analysis and correlation of *DDR1* transcripts with cell proportions

We performed a cell type enrichment analysis of each module containing *DDR1* transcripts in the HC network using the hyper() function of the hypeR package v1.14.0 [30] (Fig. 1), which performs a hypergeometric test between a reference and input lists of genes. As a reference, we retrieved a list of the top 1000 marker genes from each of the six major brain cell types (neurons, oligodendrocyte precursor cells (OPCs), oligodendrocytes, astrocytes, endothelial cells and microglia) from a meta-analysis of brain cell expression data available in the markers_df_brain() function of the BRETIGEA R package v1.0.3 [29]. The input lists corresponded to the list of the coding genes of each module. *P* values were adjusted for multiple testing [31], and an adjusted *p*-value < 0.01 was considered statistically significant. Additionally, to complement the cell type enrichment analysis, we computed the BRETIGEA cell type proportion estimates for the same cell types using the brainCells() function, setting the number of genes to the top 50 cell type-specific genes for each cell type and using the singular value decomposition approach [32]. Then, we explored the linear correlation between each relative cell type proportion estimate and the expression of individual *DDR1* transcripts (Fig. 1).

### GO enrichment and neighbor analysis of *DDR1* transcripts

We performed nonredundant Gene Ontology (GO) enrichment analyses of the biological process, molecular function and cellular components of the modules containing *DDR1* transcripts in the HC network using WebGestalt [33] (Fig. 1). All coding genes included in the study (*N* = 18163) were used as background, since they are the only genes for which GO annotation is available. *P* values were adjusted for multiple testing [31], and an adjusted *p*-value < 0.01 was considered statistically significant. Furthermore, to complement the GO enrichment analysis, we exported the topological overlap matrix of each module containing *DDR1* transcripts in the HC network, which reflects the relative interconnectedness between a pair of genes [34], into the interface of Cytoscape software to visualize the ten most correlated neighbors of each *DDR1* transcript. Nodes were colored based on their biological process as annotated in UniProt [35].

### Module preservation

We assessed the preservation of the connectivity of the HC network in SCZ and BD using the modulePreservation() function of the WGCNA R package v1.72 (Fig. 1). The degree of preservation was evaluated by means of the Zsummary, a statistic that assesses whether the connectivity level and pattern of a module in one dataset is preserved in another [27].

### Module-trait association and transcript significance

We evaluated the association of each module containing *DDR1* transcripts in the whole sample network and disease by testing the correlation between each ME and a categorical variable where HCs were assigned the value 0 and patients (SCZ and BD) were assigned a value of 1 (Fig. 1). The correlation was tested individually for patients with SCZ and patients with BD. To evaluate whether any *DDR1* transcript was independently associated with SCZ or BD, we calculated its transcript significance (Fig. 1), defined as the correlation between transcript expression and the categorical variable representing the diagnosis of SCZ or BD. P values were adjusted for multiple testing [31], and an adjusted *p*-value < 0.05 was considered statistically significant.

### Differential expression analysis

We performed a differential transcript expression analysis between patients and HCs. First, we built an expression matrix with the transcript-level counts of all genes using the readDGE() function of the edgeR package v3.40.1 [24], fitted a general linear model for each transcript and extracted the log_2_ fold change (log_2_FC) between patients and HCs (Fig. 1). SVs were included as covariates in the model. Later, to test whether each model coefficient differed significantly from zero, we performed a Wald test. The Wald test p values were subjected to independent filtering to increase the detection power [36] and were adjusted for multiple testing [31]. An adjusted *p*-value < 0.05 was considered statistically significant. The analysis was performed separately for patients with SCZ and patients with BD using the DESeq2 package v1.38.3 [37].

## Results

### Relative *DDR1* transcript expression and eQTLs

The quantification in TPM of each *DDR1* transcript included in the study is shown in Supplementary Fig. 4. ENST00000376569 (*DDR1*a), ENST00000542441 (*DDR1*b) and ENST00000376568 (*DDR1*b) were the 3 most highly expressed transcripts. After performing the eQTL analysis in the subsample of HCs (*N* = 68), we observed that some SNPs were nominally associated with the expression of *DDR1* transcripts. However, none of the associations were significant after multiple test correction; therefore, SNPs were not included as covariables in the subsequent analyses. A complete list of the nominal *p* values for each SNP is shown in Supplementary Table 1.

### Biological importance of *DDR1* transcripts

We performed WGCNA with data from HCs and detected 27 modules (Supplementary Table 2), five of which contained *DDR1* transcripts (M_HC_1- M_HC_5). The MM of *DDR1* transcripts ranged from 0.19 to 0.72 in absolute values and is shown in Table 2. Of note, five *DDR1* transcripts did not show any pattern of coexpression with other genes and were assigned to M_HC_0. To determine which cells are more likely to express specific *DDR1* transcripts, we performed a cell type enrichment analysis of each module. Three modules were enriched in astrocytes; 3 modules, in neurons; 3 modules, in oligodendrocytes; 4 modules, in endothelial cells; 4 modules, in microglia; and 6 modules, in OPCs (Supplementary Table 3). Regarding *DDR1*-containing modules, M_HC_1 was enriched in astrocytes (adjP=0.011) and OPCs (adjP=0.011); M_HC_2 was enriched in OPCs (adjP=1.3E-10); M_HC_3 was enriched in OPCs (adjP=0.0019) and oligodendrocytes (adjP=2.8E-51); M_HC_4 was enriched in OPCs (adjP=5.7E-05) and oligodendrocytes (adjP=1.7E-160); and M_HC_5 was enriched in neurons (adjP=2.3E-146). No module was enriched in microglia or endothelial cells (Table 2).

**Table 2:** Enrichment analysis of modules containing DDR1 transcripts in the HC network.

Module	*DDR1* transcripts^1^	MM	Cell-enrichment (adjP)	Biological process (enrichment ratio; adjP)
M_HC_1	ENST00000376567 (*DDR1*a) ENST00000452441 (*DDR1*b)	0.36 0.33	Astrocytes (0.011) OPCs (0.011)	Mitotic cell cycle phase transition (5.0E-04) Cilium organization (5.0E-04) Regulation of cell cycle phase transition (4.1E-03) Cell cycle G1/S phase transition (4.1E-03) Nucleic acid phosphodiester bond hydrolysis (4.6E-03) DNA conformation change (4.6E-03) Nonrecombinational repair (5.0E-03)
M_HC_2	ENST00000376570 (*DDR1*a) ENST00000324771 (*DDR1*b) ENST00000460944 ENST00000428153 ENST00000484556	0.72 0.52 0.69 0.45 −0.19	OPCs (1.3E-10)	mRNA processing ( < 2.2E-16) Ribonucleoprotein complex biogenesis ( < 2.2E-16) RNA splicing ( < 2.2E-16) ncRNA processing ( < 2.2E-16) RNA localization ( < 2.2E-16) Chromosome segregation (1.6E-09) Covalent chromatin modification (3.1E-07) Mitotic cell cycle phase transition (5.4E-07) Cell cycle checkpoint (5.4E-07) Ribonucleoprotein complex localization (8.1E-07)
M_HC_3	ENST00000508472	0.50	Oligodendrocytes (2.8E-51) OPCs (0.0019)	Establishment or maintenance of cell polarity (1.1E-03)
M_HC_4	ENST00000376568 (*DDR1*b)	0.51	Oligodendrocytes (1.7E-160) OPCs (5.7E-05)	Ensheathment of neurons (5.1E-06) Gliogenesis (5.1E-06) Peripheral nervous system development (3.4E-04)
M_HC_5	ENST00000376569 (*DDR1*a)	−0.51	Neurons (2.3E-146)	Regulation of trans-synaptic signaling ( < 2.2E-16) Regulation of ion transmembrane transport (6.3E-10) Potassium ion transport (6.3E-10) Signal release (2.3E-07) Neurotransmitter transport (2.7E-06) Cognition (6.2E-06) Regulation of neurotransmitter levels (7.2E-06) Regulation of membrane potential (1.9E-05) Vesicle-mediated transport in synapse (1.9E-05) Calcium ion regulated exocytosis (2.2E-05)
M_HC_0	ENST00000418800 (DDR1a) ENST00000513240 (DDR1c) ENST00000376575 (DDR1d) ENST00000508312 (DDR1e) ENST00000446312	NA	NA	NA

^1^The isoform encoded by each *DDR1* transcript is shown in parentheses. ENST00000460944 and ENST00000484556 encode proteins that are not classified as *DDR1*a-*DDR1*e; and ENST00000508472 is a noncoding transcript.

*adjP* *P*-value FDR-corrected, *MM* Module membership of each *DDR1* transcript in the HC network.

The correlation between *DDR1* transcript expression and the cell type proportion estimates revealed that individual *DDR1* transcripts were correlated with the cell type proportion estimates for astrocytes, OPCs, oligodendrocytes and neurons but also microglia and endothelial cells (Supplementary Fig. 5). Of note, the strongest correlations (r^2^ > 0.4 and adjP<2.2E-16) were found between *DDR1* transcripts of M_HC_2 and astrocytes, OPCs and oligodendrocytes (with the exception of ENST00000484556); of M_HC_4 and OPCs and oligodendrocytes; and of M_HC_5 and neurons. *DDR1* transcripts in M_HC_0, which did not show any pattern of coexpression with other genes and thus did not receive any cell type enrichment calculation, correlated with different cell type proportion estimates. ENST00000418800 (*DDR1*a), ENST00000508312 (*DDR1*e) and ENST00000446312 correlated mostly with neurons (adjusted *p*-values of < 2.2E-16, 1.03E-05 and 6.6E-10, respectively), and ENST00000513240 (*DDR1*c) and ENST00000376575 (*DDR1*d) correlated mostly with endothelial cells (adjusted *p* values of 5.6E-06 and 4.4E-07, respectively).

**Fig. 2:**
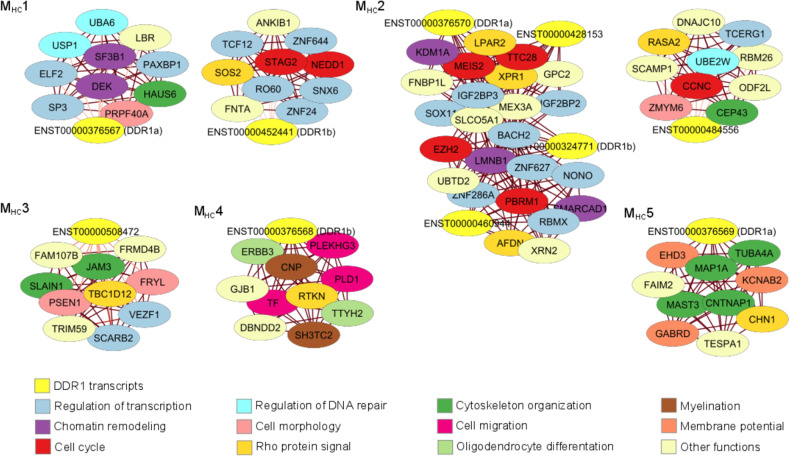
Networks of the ten more associated neighbors of each *DDR1* transcript in the HC network. Edges are colored based on the strength of association between two nodes, with darker lines representing stronger associations.

To elucidate the mechanism by which *DDR1* transcripts may be involved in these diseases, we performed a GO enrichment analysis of the modules containing *DDR1* transcripts. The results of the biological process term enrichment are provided in Table 2, and a complete list of the results is provided in Supplementary Table 4. Briefly, M_HC_1, M_HC_2 and M_HC_3 were enriched in processes that take place during cell division, M_HC_4 in gliogenesis and axon ensheathment, and M_HC_5 in synaptic signaling. To complement the GO enrichment analysis, we visualized the ten more associated neighbors of each *DDR1* transcript with Cytoscape and observed that a large proportion of them are involved in the cell cycle and processes such as regulation of transcription, chromatin remodeling and regulation of cell morphology (M_HC_1, M_HC_2, M_HC_3); oligodendrocyte differentiation and myelination (M_HC_4); and synaptic transmission (M_HC_5) (Fig. 2).

### *DDR1* transcript module preservation and association with SCZ and BD

**Fig. 3:**
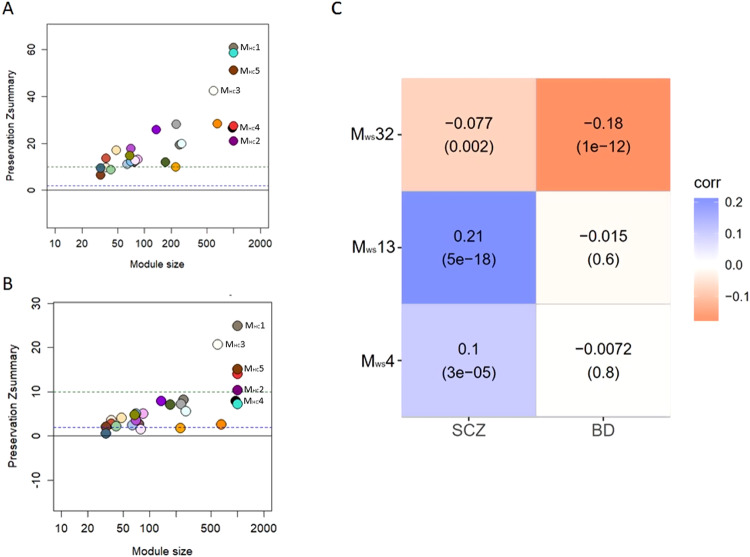
*DDR1* transcript module preservation and association with disease. **A**, **B** Depict the results of the module preservation test of the SCZ (**A**) and BD (**B**) networks. Modules containing *DDR1* transcripts are colored as follows: M1=Saddle brown, M2=Dark magenta, M3=Antique white, M4=Black, M5=Brown. Zsummary < 2 indicates lack of preservation; 2 ≤ Zsummary ≤ 10, weak preservation; Zsummary > 10, strong preservation. (**C**) is a heatmap of the association of each DDR1 transcript module with the diagnoses of SCZ and BD in the whole sample network.

We assessed whether there was any gene coexpression disruption in the SCZ and BD networks with respect to the HC network by calculating the Zsummary of each module containing *DDR1* transcripts. All modules containing *DDR1* transcripts were preserved in both SCZ and BD (Fig. 3A, B), so we used the whole dataset to evaluate the association of each module with SCZ and BD [38]. Twenty-nine modules were detected in the whole dataset. Eight *DDR1* transcripts were scattered across three modules (M_ws_4, M_ws_13 and M_ws_32), and seven *DDR1* transcripts did not show any pattern of coexpression with other genes and were assigned to M_ws_0. The MM of *DDR1* transcripts ranged from 0.11 to 0.68 and is detailed in Supplementary Table 5. M_ws_4 contained ENST00000376575 (*DDR1d*) and ENST00000452441 (*DDR1b*) and was associated with SCZ and BD. M_ws_13 contained ENST00000460944 and was associated with SCZ. M_ws_32 contained ENST00000376567 (*DDR1a*), ENST00000376570 (*DDR1a*), ENST00000324771 (*DDR1*b), ENST00000428153 and ENST00000446312 and was associated with SCZ and BD (Fig. 3C and Supplementary Table 5). Of note, the MEs of M_ws_4, M_ws_13 and M_ws_32 were correlated with the cell type proportion estimates of the normalized whole sample matrix (Supplementary Table 6), which indicates that the variance explained by the cell type composition of the samples was not completely removed when building the network.

### *DDR1* transcript differential expression analysis and transcript significance in SCZ and BD

**Fig. 4:**
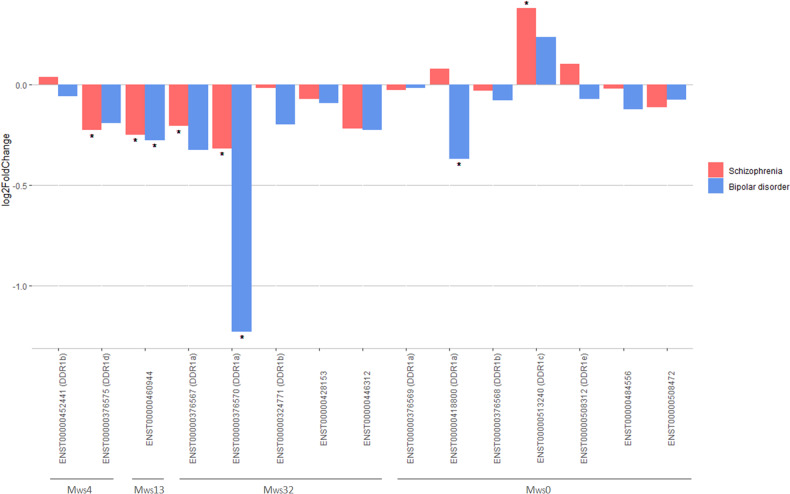
Differential transcript expression analysis. *DDR1* transcripts are distributed along the x-axis and grouped by their module in the whole sample network (M_ws_). Asterisks indicate statistically significant associations with schizophrenia (coral bars) and bipolar disorder (blue bars).

We observed that some *DDR1* transcripts present in M_ws_4, M_ws_13 and M_ws_32 were differentially expressed in SCZ or BD (Fig. 4). ENST00000376575 (*DDR1*d) was downregulated in SCZ (log_2_FC = −0.21; adjP = 0.031), ENST00000460944 was downregulated in SCZ (log_2_FC = −0.25; adjP = 0.0012) and BD (log_2_FC = −0.28; adjP = 0.028), ENST00000376567 (*DDR1*a) was downregulated in SCZ (log_2_FC = −0.23; adjP=0.048), ENST00000376570 (*DDR1*a) was downregulated in SCZ (log_2_FC = −0.32; adjP=0.037) and BD (log_2_FC = −1.23; adjP = 3.2E-08), ENST00000418800 (*DDR1*a) was downregulated in BD (log_2_FC = −0.37; adjP = 0.015) and ENST00000513240 (*DDR1*c) was upregulated in SCZ (log_2_FC = 0.34; adjP = 0.0062). These associations were further validated by the transcript significance test, which showed that all transcripts differentially expressed in SCZ or BD were significantly correlated in the same direction with the same diagnosis (Supplementary Table 5). Although ENST00000376567 (*DDR1*a), ENST00000376575 (*DDR1*d) and ENST00000513240 (*DDR1*c) did not reach statistical significance in the differential expression analysis for BD after adjusting for multiple testing, they followed the same trend as in SCZ (Fig. 4), and they were significantly correlated with BD in the transcript significance test (Supplementary Table 5).

## Discussion

**Fig. 5:**
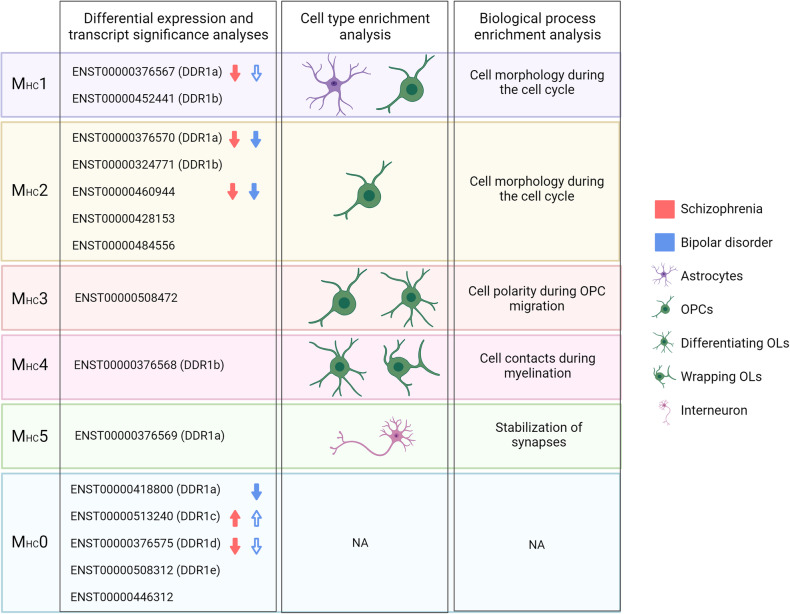
Involvement of *DDR1* transcripts in SCZ and BD Arrows pointing up and down represent *DDR1* transcripts upregulated and downregulated in disease, respectively. Filled arrows indicate *DDR1* transcripts with a *p*-value < 0.05 in the differential transcript expression and in the transcript significance analyses. Transparent arrows represent *DDR1* transcripts with a *p*-value < 0.05 in the transcript significance test. Colors represent the disorders. OLs Oligodendrocytes, OPCs Oligodendrocyte precursor cells. Figure created with BioRender.com.

Here, we studied *DDR1* transcript coexpression in the adult human brain using transcriptomic data for the first time. Using expression data from HCs, we observed that coexpression modules including *DDR1* transcripts were enriched in marker genes for astrocytes, OPCs, oligodendrocytes and neurons. Using expression data from patients with SCZ, patients with BD and HCs, we observed that all coexpression modules containing *DDR1* transcripts were associated with SCZ or BD and that six *DDR1* transcripts were differentially expressed in SCZ or BD (Fig. 5).

*DDR1* transcripts in M_HC_1 (ENST00000376567 (*DDR1*a) and ENST00000452441 (*DDR1*b)) and M_HC_2 (ENST00000376570 (*DDR1*a), ENST00000324771 (*DDR1*b), ENST00000460944, ENST00000428153 and ENST00000484556) were coexpressed with genes involved in processes linked to the cell cycle, DNA repair and RNA processing, as revealed by GO enrichment analysis of the modules, which suggests that they may be expressed during cell division and proliferation. Cell enrichment analysis of the modules and correlation of *DDR1* transcripts with cell type proportion estimates suggested that the expression of *DDR1* transcripts in M_HC_1 is not restricted to a single cell type and that they are probably expressed in astrocytes and OPCs. Conversely, *DDR1* transcripts in M_HC_2 are hub transcripts of the module and therefore could be specifically expressed in OPCs. An exception in M_HC_2 is ENST00000484556, which does not represent a hub transcript, forms a different cluster in the network visualization analysis and correlates mostly with neuron proportion estimates. Regarding the possible role of *DDR1* transcripts in cell division and proliferation, previous research showed that *DDR1* is recognized and upregulated by the p53 protein [17, 39]. p53 activates the checkpoint needed to avoid DNA damage accumulation during the cell cycle G1/S phase transition [40] and mediates cell prosurvival effects [39]. Accordingly, M_HC_1 and M_HC_2 are enriched in mitotic and/or cell cycle G1/S phase transition genes and include genes that induce the p53 cascade, such as *TP53INP1*, *TP53BP1*, *TP53I3*, *TP53RK* and *TP53I11*. Moreover, *DDR1* has been reported to be involved in actin cytoskeleton remodeling [41], and *DDR1* transcripts in M_HC_1 and M_HC_2 were coexpressed with genes involved in cell morphology and cytoskeleton organization in the network visualization analysis. It is thus possible that p53 upregulates these *DDR1* transcripts during the cell cycle to ensure the maintenance of cell morphology in different cell types.

*DDR1* transcripts in M_HC_3 (ENST00000508472) and M_HC_4 (ENST00000376568 (*DDR1*b)) could be expressed from late stages of OPC differentiation to early stages of oligodendrocyte differentiation/maturation, since they are hub transcripts of M_HC_3 and M_HC_4, which are enriched in OPCs and oligodendrocytes. Additionally, ENST00000376568 (*DDR1*b) correlates mostly with the OPC and oligodendrocyte proportion estimates. ENST00000508472 could be involved in the establishment of cell polarity during OPC migration, given the enrichment in the establishment or maintenance of cell polarity of M_HC_3, the coexpression with genes devoted to cell morphology and cytoskeleton organization in the network visualization analysis and the fact that ENST00000508472 is expressed at low levels, consistent with the low rate of OPC migration in adulthood [42]. ENST00000376568 (*DDR1*b) is coexpressed with genes upregulated in the final stage of newly formed oligodendrocytes and early stages of mature oligodendrocytes [43] and was previously observed to be locally translated in oligodendrocyte processes to synthesize myelin (*MBP*, *MOBP*, *BCAS1* and *PLEKHB1* [44, 45]). We previously demonstrated that *DDR1* has an A2 response element (A2RE) sequence that is recognized by heterogeneous nuclear ribonucleoprotein (hnRNP) A2/B1, and silencing the hnRNP A2/B1 gene in an oligodendroglial cell line resulted in a downregulation of DDR1c concomitant with an upregulation of DDR1b [20]. Since ribonucleoproteins are involved in RNA‒protein packaging and transport from the nucleus to the cytoplasm periphery, we can speculate that ENST00000376568 (*DDR1*b) is the main isoform that is involved in myelination. Accordingly, previous studies have shown that *DDR1* parallels the dynamics of myelination [9], is important for the compactness of the myelin sheath [46] and regulates the ensheathment, survival and caliber of peripheral axons [47]. Furthermore, *DDR1* stabilizes cadherins in cell membranes [48, 49]. Here, module M_HC_4 contains the cadherin *CDH19*, a marker of myelin-forming cells [50]. Altogether, our results suggest that ENST00000376568 (*DDR1*b) can stabilize cadherin contacts between oligodendrocyte and neuron membranes or, alternatively, between concentric oligodendrocyte membranes required for compacting the myelin sheath, as previously suggested [22, 46]. In addition, the expression of ENST00000376568 (*DDR1*b) in oligodendrocytes is supported by single-cell transcriptome studies showing that *DDR1* is mainly expressed in oligodendrocytes in the human brain (www.proteinatlas.org).

The *DDR1* transcript of M_HC_5 (ENST00000376569 (*DDR1*a)) is a hub transcript that may be expressed in interneurons and contribute to synapse stabilization, since it is highly enriched in neurons and processes related to synapses and contains most of the classical interneuron genes, namely, *SST*, *CCK*, *GAD1*, *PVALB*, *CALB1*, *VIP*, *LAMP5*, *RELN* and *NOS1* [51]. Accordingly, the expression of *DDR1* is higher in inhibitory than in excitatory neurons (www.proteinatlas.com). Recent research reported the expression of *DDR1* in mature GABA neurons of the mouse adult brain [52], and some studies aimed at the characterization of the expression profile of the different types of interneurons have detected a slight expression of *DDR1* across all types of interneurons, especially in those expressing *SST* or *CCK* [53, 54]. Interneurons are enfolded by perineuronal nets, a specialization of the extracellular matrix required for controlling the plasticity of the central nervous system [55]. Interestingly, *DDR1* binds some types of collagens, important constituents of the extracellular matrix [56], and collagen XIX has been demonstrated to be pivotal for the stability of perineuronal nets and synapse formation [57, 58]. To the best of our knowledge, the binding of *DDR1* to collagen XIX has not yet been studied, but the collagen XIX gene (*COL19A1*) is located in M_HC_5. Altogether, we hypothesize that ENST00000376569 (*DDR1*a) may bind to some components of the perineuronal net, leading to the stabilization of newly formed synapses.

*DDR1* transcripts of M_HC_0 could not be assigned a function by enrichment analyses. Among them, we observed that the expression of ENST00000418800 (*DDR1*a), ENST00000508312 (*DDR1*e) and ENST00000446312 correlated mostly and negatively with the cell proportion estimates of neurons and that ENST00000513240 (*DDR1*c) and ENST00000508312 (*DDR1*e) correlated mostly with the cell proportion estimates of endothelial cells. However, the correlation coefficients are low ( < 0.4), which precludes the assignment of these transcripts to a specific cell type. According to the present results, we previously reported that the average methylation level of *DDR1* in isolated neuronal nuclei correlated negatively with the average methylation level of neuron markers such as *MAP2* and *MAPT* [59]. In addition, in a previous study, we detected the expression of *DDR1* in brain tissue endothelial cells by means of in situ hybridization and immunohistochemistry. Specifically, we detected signals using antibodies against the *DDR1* extracellular domain but not with antibodies against the intracellular domain [12]. This observation is consistent with the fact that ENST00000376575 (*DDR1*d), which lacks the intracellular domain, correlates with the cell proportion estimates of endothelial cells.

The expression of *DDR1* in activated microglia has been reported in some studies [60, 61]. Using coexpression modules in different stages of human brain development, we observed that the expression of *DDR1* in microglia was relevant in the prenatal period, the first years of life ( < 6 years) and late adulthood ( ≥ 40 years), but not the period between 7 and 40 years of age [23]. Here, we found that some transcripts correlated with the cell proportion estimates of microglia, especially ENST00000376570 (*DDR1*a), which showed a negative correlation, in agreement with our previous observation that the average methylation level of *DDR1* in brain tissue correlated negatively with the average methylation level of the microglia marker *CX3CL1* [59]. However, in the present study the correlation coefficient was not sufficiently high ( < 0.4) to draw conclusions about the relevance of this transcript in microglial function.

Regarding the involvement of *DDR1* in psychiatric disorders, our results indicate that *DDR1* transcript expression is more altered in SCZ than in BD. All modules containing *DDR1* transcripts in the whole sample network (M_ws_4, M_ws_13 and M_ws_32) were associated with SCZ, while only M_ws_32 was associated with BD. However, it is worth noticing that the MEs of M_ws_4, M_ws_13 and M_ws_32 were correlated with cell-type proportion estimates of many cell types, which could partly explain the associations of M_ws_4, M_ws_13 and M_ws_32 with the diagnoses of SCZ and BD. On the other hand, he expression of 6 transcripts was altered in the differential expression and transcript significance analyses in SCZ, while that of only 3 transcripts was altered in the transcript significance test in BD (Fig. 5). According to our interpretation, some transcripts downregulated in psychiatric disorders (ENST00000376567 (*DDR1*a), ENST00000376570 (*DDR1*a), ENST00000460944) may be involved in the maintenance of the cell morphology of astrocytes and OPCs during the cell cycle. The only transcript that was upregulated in disease was ENST00000513240 (*DDR1*c), in agreement with previously published evidence that *DDR1*c is upregulated in patients with SCZ [21]. In addition, the transcript ENST00000418800 (*DDR1*a) was downregulated only in BD. Whether alterations in the expression of *DDR1* transcripts are a cause or a consequence of the disease cannot be inferred from our results. However, according to previous evidence, the upregulation of ENST00000513240 (*DDR1*c) may be at least partly attributable to genetic variation, since minor alleles of rs2267641 and rs1264323 are associated with SCZ diagnostic and cognition speed processing [4, 62] and with higher levels of ENST00000513240 (*DDR1*c) in SCZ [22]. Overall, our study suggests that patients with SCZ and BD present a downregulation of *DDR1*a during the cell cycle of astrocytes and OPCs and proposes that the expression of other *DDR1* transcripts is also altered in other cell types. This evidence supports the growing awareness that the cell type-specific regulation system should be considered in order to disentangle the complex physiopathology of SCZ and BD [63, 64], which could help the development of more specific therapies based upon the targeting of specific cells, a promising strategy currently in development in other fields [65]. We hope that future research using single-cell transcriptomic data will validate the results presented here.

## Limitations

The present study has some limitations that are worth mentioning. First, our results do not represent the direct measurement of mRNA in single cells but rather the indirect inference of single-cell mRNA expression based on the principle of “guilt by association” [27]. Second, a shared pattern of expression does not necessarily mean physical proximity, and we did not perform in situ experiments to confirm the pattern of *DDR1* isoform expression across cell types. Third, our analyses only considered the six major types of brain cells, while there is a growing trend toward cell subtype classification of brain cells [43, 52, 66]. Fourth, our results are based on samples from the dorsolateral prefrontal cortex, and different results may be found in other brain regions [67]. Fifth, many *DDR1* transcripts were filtered out at the beginning of the analysis, and thus, the landscape of *DDR1* expression and function was not fully addressed. Sixth, the results of the differential expression analysis should be replicated in independent samples.

### Supplementary information

Supplementary Material

Supplementary Figure 1

Supplementary Figure 2

Supplementary Figure 3

Supplementary Figure 4

Supplementary Figure 5

Supplementary Tables

## Data Availability

Data used in this manuscript was retrieved from PsychENCODE and no new data was generated.

## Code availability

Code is available upon request from the corresponding author.
