# Supplementary material for: Coexpression network analysis of the adult brain sheds light on the pathogenic mechanism of DDR1 in schizophrenia and bipolar disorder
Source: Transl Psychiatry. 2024 Feb 23;14:112. doi: 10.1038/s41398-024-02823-0 (PMC10891045; doi:10.1038/s41398-024-02823-0)
Supplement: Supplementary file 1 — Supplementary Material [file 41398_2024_2823_MOESM1_ESM.docx]

**Supplementary Material**

Expression data

The expression data used for this manuscript are derived from the PsychENCODE consortium and are available at <http://www.doi.org/10.7303/syn12080241>. The dataset consists of gene and transcript-level counts from dorsolateral prefrontal cortex samples obtained by RNA-Seq by Expectation-Maximization from six different studies, which are described below.

*BrainGVEX*

We included RNA-Seq data generated from healthy controls (N=94), patients with schizophrenia (N=94) and patients with bipolar disorder (N=73). RNA was extracted using the Lysin Matrix D and FastPrep-24 system (MP Biomedicals). RNA integrity was assessed by the Agilent Technologies RNA 600 Nano kit, and only samples with an RNA integrity number (RIN) above 5.5 were included. RNA sequencing libraries were prepared using the TruSeq Stranded Total RNA sample prep kit with RiboZero Gold HMR (Illumina), and libraries were multiplexed (3 per lane) for paired-end 100 bp sequencing on Illumina HiSeq2000 with read depth >70 million reads on average.

*BrainSpan*

RNA-Seq from 22 healthy controls was included in our analysis. RNA was extracted using the RNeasy Plus Mini Kit (Qiagen) for mRNA. The RIN was determined for each sample with an Agilent 2100 Bioanalyzer system, and only samples with an RIN >6 were included in the study. cDNA libraries were prepared using the Illumina mRNA-sequencing (mRNA-Seq) sample Kit according to the manufacturer’s instructions, followed by PCR amplification. The PCR-enriched product was assessed for its size distribution and concentration using a Bioanalyzer DNA 1000 Kit.

*CMC*

We included RNA-Seq data generated from healthy controls (N=284), patients with schizophrenia (N=264) and patients with bipolar disorder (N=47). RNA was extracted using the RNeasy kit, and only samples with an RIN >5.5 were included. RNA-Seq library preparation was performed using ribosomal RNA depletion with the RiboZero Magnetic Gold Kit. Samples were multiplexed (10 per line) for paired-end 100 bp sequencing on an Illumina HiSeq2000 instrument and yielded 85 million reads on average.

*BipSeq*

RNA-Seq data from 32 patients with bipolar disorder were included in our analysis. RNA was extracted using the RNeasy kit (Qiagen), and samples with an RIN >5 were included. RNA-Seq libraries were constructed using the Illumina TruSeq RNA Sample Preparation v2 kit. Libraries were multiplexed for paired-end 100 bp sequencing on an Illumina HiSeq2000 instrument.

*LIBD_szControl*

We included RNA-Seq data generated from healthy controls (N=151) and patients with schizophrenia (N=108). RNA was extracted using the RNeasy kit (Qiagen), and samples with an RIN >5 were included. RNA-Seq libraries were constructed using the Illumina TruSeq RNA Sample Preparation v2 kit. Libraries were multiplexed for paired-end 100 bp sequencing on an Illumina HiSeq2000. We also used genotyping data from 68 healthy controls. Genotyping was performed using HumanHap650Y_V3, Human 1M-Duo_V3 and Omni5 BeadChips (Illumina) according to the manufacturer’s instructions. Genotype data were processed and normalized with the crlmm R package separately by platform.

CMC_HBCC

We included RNA-Seq data generated from healthy controls (N=220), patients with schizophrenia (N=97) and patients with bipolar disorder (N=222). RNA was extracted using the RNeasy kit (Qiagen), and samples with an RIN >5.5 were included. Libraries were prepared using the KAPA Stranded RNA-Seq Kit with RiboErase (KAPA Biosystems) and subsequently sequenced on an Illumina HiSeq2000 instrument.
