# Supplementary figures and images for: Coexpression network analysis of the adult brain sheds light on the pathogenic mechanism of DDR1 in schizophrenia and bipolar disorder
Source: Transl Psychiatry. 2024 Feb 23;14:112. doi: 10.1038/s41398-024-02823-0 (PMC10891045; doi:10.1038/s41398-024-02823-0)

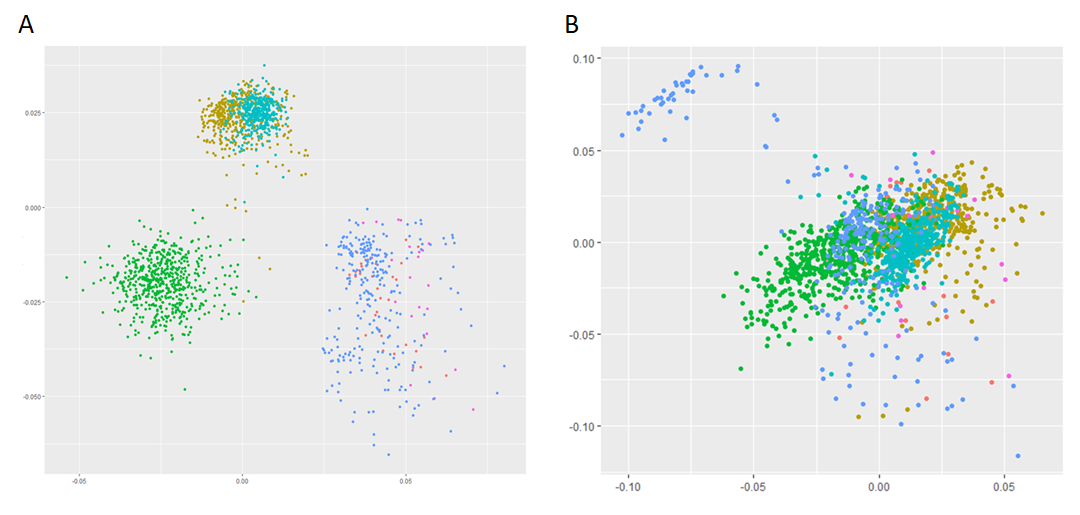

Supplement: Supplementary file 2 — Supplementary Figure 1 [file 41398_2024_2823_MOESM2_ESM.png]

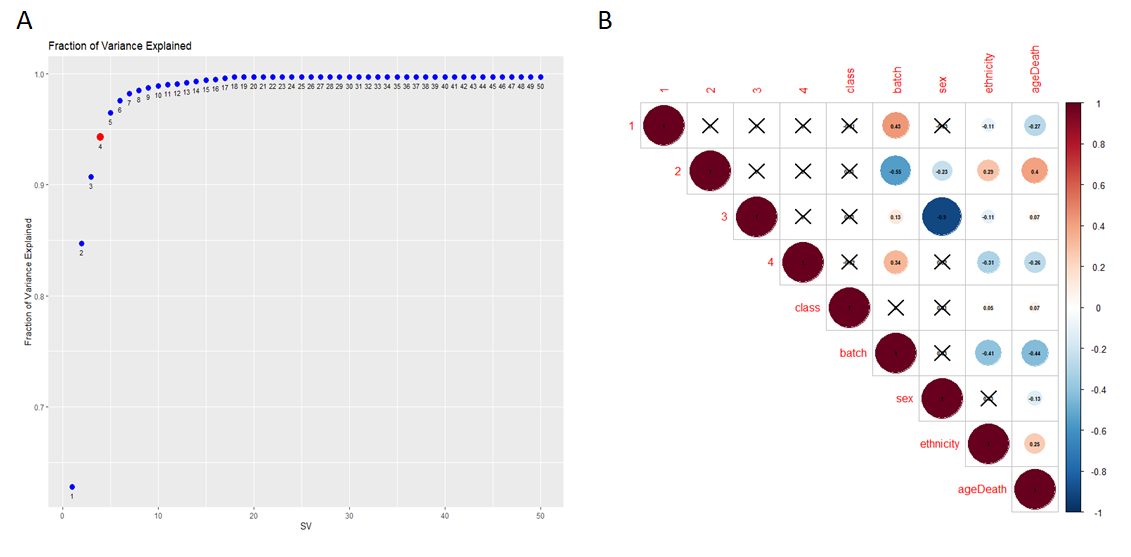

Supplement: Supplementary file 3 — Supplementary Figure 2 [file 41398_2024_2823_MOESM3_ESM.png]

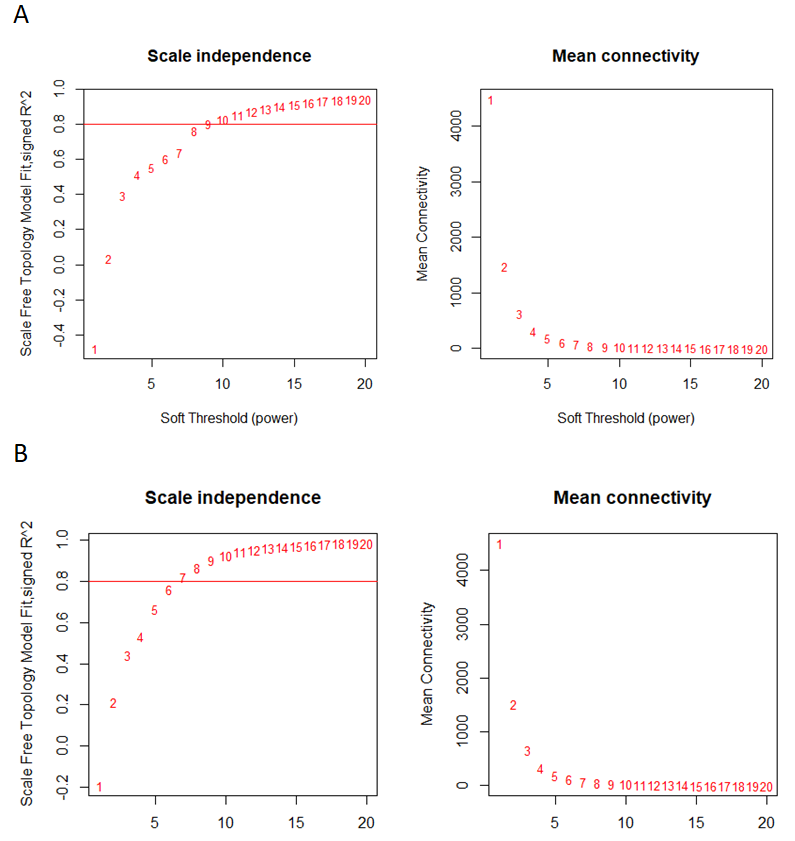

Supplement: Supplementary file 4 — Supplementary Figure 3 [file 41398_2024_2823_MOESM4_ESM.png]

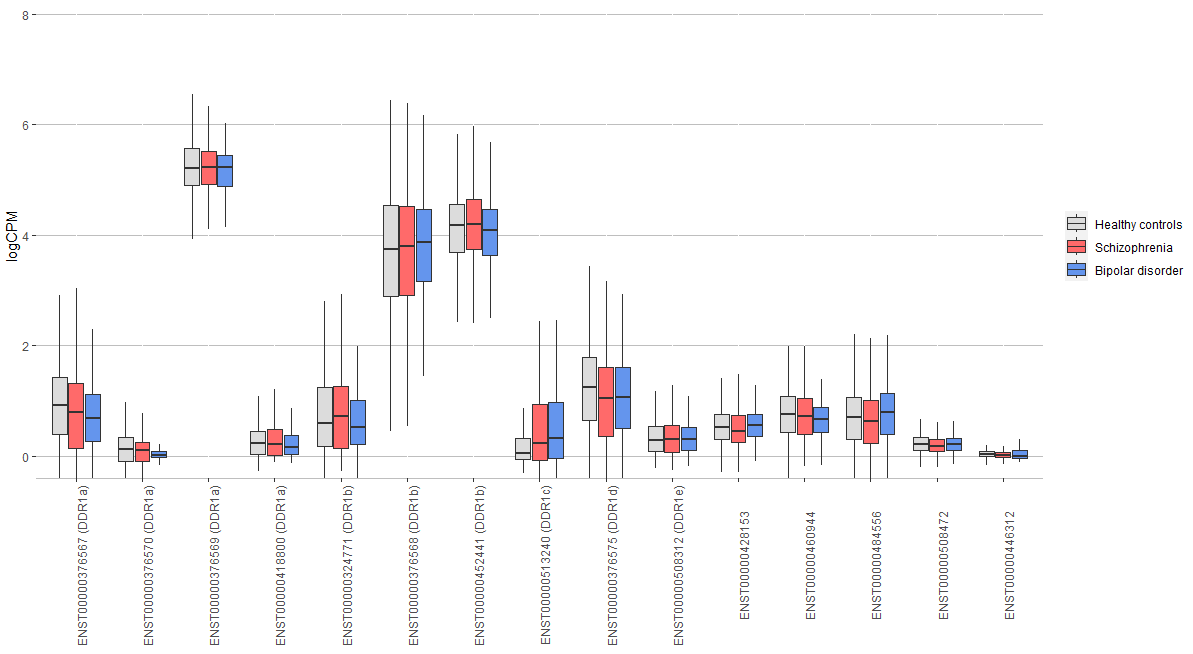

Supplement: Supplementary file 5 — Supplementary Figure 4 [file 41398_2024_2823_MOESM5_ESM.png]

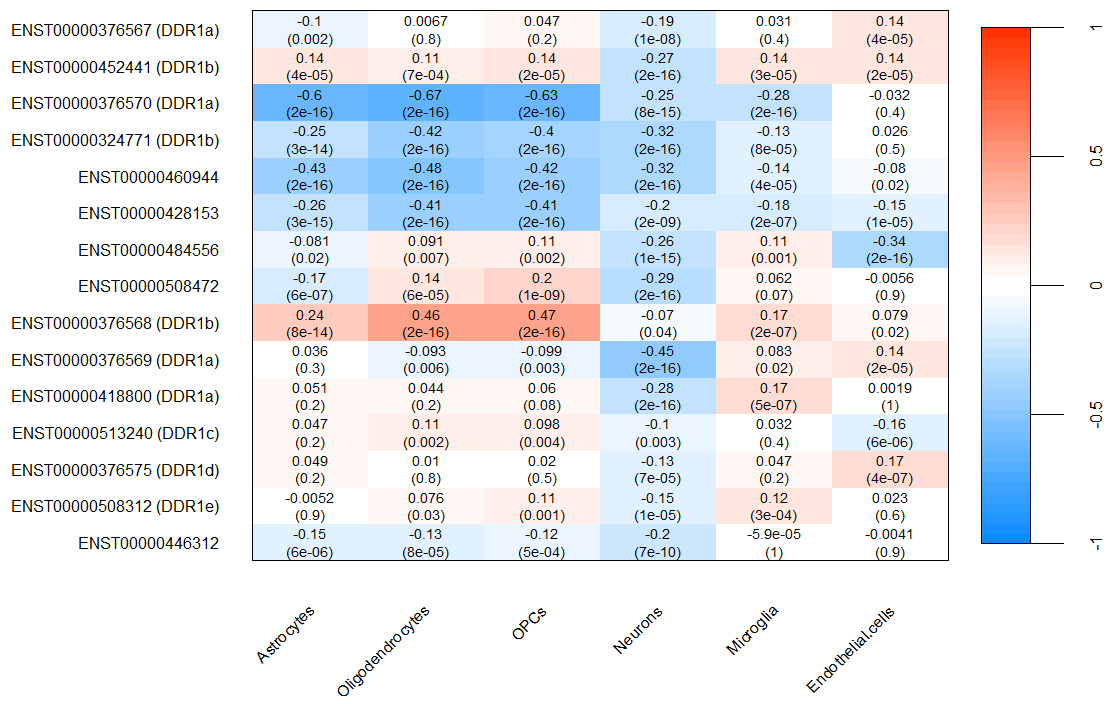

Supplement: Supplementary file 6 — Supplementary Figure 5 [file 41398_2024_2823_MOESM6_ESM.png]
